# Targeting Yeast Pathogens with Lectins: A Narrative Review from Mechanistic Insights to the Need for Addressing Translational Challenges

**DOI:** 10.3390/biomedicines14010105

**Published:** 2026-01-05

**Authors:** Gustavo Ramos Salles Ferreira, Thiago Lucas da Silva Lira, Thiago Henrique Napoleão

**Affiliations:** Departamento de Bioquímica, Centro de Biociências, Universidade Federal de Pernambuco, Recife 50670-901, Brazil; gustavo.rsferreira@gmail.com (G.R.S.F.); thiago.silvalira@ufpe.br (T.L.d.S.L.)

**Keywords:** *Candida*, *Cryptococcus*, antimicrobial activity

## Abstract

Diseases associated with yeast pathogens have become an increasingly serious global health issue. The range of virulence factors and the development of mechanisms of resistance have posed a significant challenge in the fight against these types of infections. Lectins, proteins capable of reversibly binding to carbohydrates and glycoconjugates, have been assessed as antifungal agents. This review shows that lectins have demonstrated versatility and significant potential as therapeutic agents against *Candida*, *Nakaseomyces* and *Cryptococcus*. These molecules act through diverse mechanisms, including disruption of fungal cell membranes, induction of oxidative stress, inhibition of ergosterol biosynthesis, and interference with mitochondrial and lysosomal functions. Some lectins have been shown to inhibit yeast-to-hyphae morphological transitions and biofilm formation, which are critical virulence factors for pathogenic yeasts. Moreover, some lectins have shown potential to enhance the efficacy of conventional antifungal drugs through synergistic interactions, though these effects can depend on the fungal isolate. Beyond in vitro activity, translational considerations remain underdeveloped in the context of antifungal applications of lectins. Some lectins exhibit minimal toxicity, while others require careful dosing due to potential toxicity or undesired immunogenicity. Delivery and stability also present challenges, though strategies such as chemical modifications and topical, mucosal, or nanoparticle-based formulations show promise. Overall, the multifaceted antifungal activities of lectins highlight their promising role as innovative candidates in the development of novel therapies to address the growing challenge of yeast pathogen resistance. However, significant knowledge gaps persist, highlighting the urgent need for coordinated research that bridges in vitro findings with practical pharmacological applications.

## 1. Introduction

Fungal infections are an increasingly serious global health threat, affecting an estimated 150 million people annually and causing approximately 1.7 million deaths [1]. Delayed diagnosis and late initiation of appropriate treatment are key factors contributing to the high morbidity and mortality associated with invasive fungal infections [2]. These challenges, combined with the long-term and widespread use of antifungal medications—particularly among high-risk groups such as the elderly, pregnant women, children, and immunocompromised individuals—have contributed to the emergence of multidrug-resistant strains [1,3].

Fungal infections are presently managed using four primary categories of antifungal medications: azoles, echinocandins, polyenes, and pyrimidine analogs. However, resistance mechanisms to these antifungals are diverse. They include overexpression of target proteins (azoles); mutations in target proteins (azoles and echinocandins); increased production and/or membrane insertion of efflux pumps (azoles); and limited access to drug targets, such as ergosterol sequestration (polyenes) [4]. In addition, fungal virulence factors (secretion of hydrolytic enzymes and biofilm formation) play a crucial role in both the progression of infection and fungal tolerance to conventional antifungal agents [2,5,6,7]. As a result, there is a growing interest in antifungal agents derived from natural sources, such as lectins.

**Figure 1:**
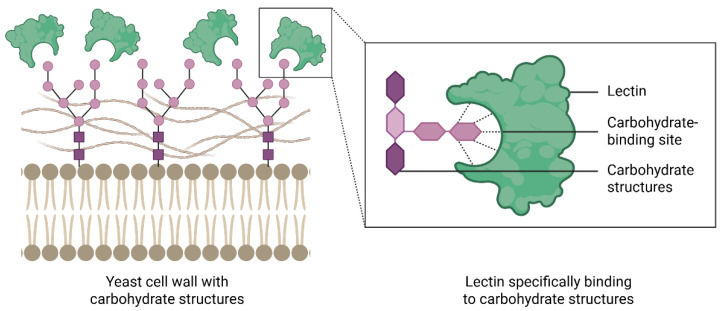
Lectin recognition of carbohydrate structures on the yeast cells. Lectins can bind specifically and reversibly to glycan residues on the fungal cell surface through their carbohydrate binding sites, mediating interactions with mono-, oligo-, polysaccharides and glycoproteins. This specific molecular recognition underlies diverse biological processes, including cell signaling, adhesion, and antimicrobial activity. Created in BioRender. Lira, T.L.S. (2025) https://BioRender.com/p48asim.

Lectins are proteins widely distributed in nature and are found in microorganisms, plants, and animals. They possess at least one non-catalytic site that allows reversible binding to mono-, oligo-, or polysaccharides (Figure 1). The carbohydrate-binding properties of lectins enable interactions with cells and play important roles in cellular processes. Due to these properties, lectins exhibit a wide range of biological activities, including immunomodulatory [8], antiviral [9], insecticidal [10], anticancer [11], and anti-inflammatory [12] effects, among others.

The antimicrobial activity of lectins occurs through interactions with components of microbial surface, including peptidoglycans, lipopolysaccharides, teichoic and teichuronic acids, glucans, mannans, and chitin. These interactions can lead to alterations in cell permeability, pore formation on the microbial surface, agglutination of microbial cells, inhibition of growth, and ultimately, cell death [13]. Lectins are also recognized as potent natural agents with antibiofilm activity by disrupting the polymerization of key components involved in biofilm formation as well as interfering with biofilm development by reducing microbial cell viability, downregulating genes related to quorum sensing, and interacting with surfactants, enzymes, and polysaccharides essential to biofilm structure [14]. Due to these mechanisms of action, lectins have attracted increasing interest as potential antifungal agents against pathogenic yeasts.

In 2020, Del Rio et al. [15] reviewed the antifungal potential of lectins against human pathogens, summarizing both their lethal and non-lethal effects as well as the mechanisms underlying these activities. Over the subsequent five years (2021–2025), a significant body of new research has deepened our understanding of the cellular and subcellular effects of lectins on yeasts. Additionally, studies have provided growing evidence of the efficacy of lectins against *Cryptococcus* species and have explored their synergistic interactions with conventional antifungal agents. As research on the antifungal activity of lectins against yeasts expands, there is a rising need to investigate translational aspects of this approach, including preclinical evaluations of toxicity, efficacy, and potential pharmaceutical strategies.

This narrative review was conducted by searching for scientific articles across multiple databases, including PubMed, Springer Link, National Center for Biotechnology Information (NCBI), ScienceDirect, Wiley Online Library, Scopus, Google Scholar, and Scielo. The initial search used the descriptors “lectin” and “antifungal activity” without temporal restrictions, and only studies evaluating yeast pathogens were selected. In a second step, articles addressing the toxicity of the lectins reported to be active against yeasts were identified using the terms “toxicity” or “safety” combined with the specific lectin name. To explore other translational aspects, additional research was performed using relevant terms encompassing lectin names and formulations and drug delivery strategies. In addition, reviewing the discussion sections of the selected papers led to the identification and inclusion of additional relevant articles.

Accordingly, this review provides an updated assessment of the current state of knowledge on the antifungal activity of lectins against yeast pathogens of the *Candida*, *Nakaseomyces* and *Cryptococcus* genera, which encompass the species for which these proteins have demonstrated activity. It also examines critical considerations for their potential application, including toxicity, immunogenicity, selectivity, and delivery strategies, highlighting the urgent need for studies addressing translational challenges.

## 2. Pathogenic Yeasts: *Candida* and *Cryptococcus*

The genus *Candida* comprises a group of ascomycete fungi with a yeast-like morphology that are widely distributed in nature. These organisms typically live saprophytically as part of the normal human microbiota; however, under certain host conditions, they can transition from a commensal to a pathogenic form, leading to opportunistic infections known as candidiasis [16]. Candidiasis may present in superficial forms—such as infections of the skin and mucous membranes—or in systemic (invasive) forms, which can affect various organs and tissues. Although systemic candidiasis is less common than superficial infections, it is associated with a high mortality rate, ranging from 35% to 80% depending on the *Candida* species [17].

The most prevalent pathogenic species within the *Candida* genus is *Candida albicans*, accounting for approximately 50% of infections in the United States and Europe. This is followed by *C. parapsilosis*, *C. tropicalis*, and *C. krusei*. It is also important to highlight that *Nakaseomyces glabratus* was listed in the second position in the study, as it was referred to as *Candida glabrata* at the time [18]. Despite its lower overall prevalence, *Candida auris*, a highly drug-resistant species, has emerged as a major cause of hospital-acquired infections worldwide [19].

Although most infections caused by *Candida* species can be treated with antifungal agents such as azoles, polyenes, echinocandins, and 5-fluorocytosine, several resistance mechanisms to these drugs have been identified. These include mutations in the ergosterol biosynthesis pathway, overexpression of efflux pumps, mutations affecting cell wall biosynthesis, and alterations in nucleic acid synthesis or repair genes [20]. Additionally, the ability of *Candida* species to form biofilms further contributes to antifungal resistance. Biofilms provide both physical and molecular protection, reducing drug penetration and efficacy, while also promoting the dissemination of the pathogen to other regions of the host, thus posing an even greater health risk [18,21].

The cell wall of *Candida albicans* is a two-layered structure. The inner layer is composed of a β-glucan and chitin skeleton. β-1,3-glucans are the most abundant molecules in the inner layer and are linked to β-1,6-glucans, which function as connectors between the inner and outer layers. The outer layer is rich in mannoproteins cross-linked to β-1,6-glucans [22].

*Cryptococcus* is a genus of basidiomycete fungi with a yeast-like morphology and predominantly aerobic metabolism. These fungi inhabit a wide variety of environments and are characterized phenotypically by the presence of a polysaccharide capsule surrounding their cells [23,24,25]. *Cryptococcus neoformans* and *Cryptococcus gattii* are the primary species of clinical concern, as they are opportunistic pathogens capable of causing cryptococcosis, an invasive infectious disease that primarily affects immunocompromised patients and targets the central nervous system. This fungal meningoencephalitis is associated with a high mortality rate worldwide, in both developed and developing countries [23,26].

The main virulence factors of *Cryptococcus* include the exopolysaccharide capsule, which not only protects the fungal cell from the host immune system but also contributes to its virulence compounds. Other factors are melanin production (a dark pigment that confers resistance to multiple stressors such as elevated temperatures, free radicals, and ionizing radiation) as well as biofilm formation [24,27].

Treatment for cryptococcosis varies according to infection severity and the host’s immune status. *Cryptococcus* species are generally susceptible to three classes of antifungal agents: azoles, fluorocytosine, and polyenes [23,28]. However, resistant strains to fluorocytosine [29], fluconazole [30], and amphotericin B [28] have been reported.

The cell wall of *C. neoformans* is a dynamic two-layered structure that undergoes constant remodeling. The inner layer is composed of β-glucans and chitin arranged as fibers parallel to the plasma membrane, while the outer layer contains α-1,3-glucan and β-glucan. β-1,6-Glucan is the most abundant component of the cell wall. Chitin is present in smaller amounts and chitosan is also part of the cell wall. Additionally, the wall contains mannoproteins. The exopolysaccharide capsule (composed of glucuronoxylomannan, galactoxylomannan and mannoproteins) is anchored to the outer layer of the cell wall, and this attachment is crucial for its function [22,25].

In this scenario, natural products may offer novel mechanisms of action capable of overcoming resistance, thereby improving the effectiveness of treatments against infections caused by *Candida* and *Cryptococcus* species. Because yeast cell walls and membranes are rich in carbohydrate components, lectins have been investigated as potential antifungal agents against these pathogens.

## 3. Antifungal Activity of Lectins on *Candida* Species

Lectins have demonstrated significant activity against clinically relevant *Candida* strains. Table 1 summarizes examples of lectins with antifungal activity against *Candida* spp. and *N. glabratus,* detailing their carbohydrate-binding specificity, experimental protocols, minimal inhibitory concentrations (MIC), minimal fungicidal concentrations (MFC), and observed effects. The studies report that they exhibit inhibitory and/or fungicidal effects by compromising fungal cell wall and membrane integrity, inducing oxidative stress, disrupting mitochondrial function, depleting ATP, promoting apoptosis, and causing DNA damage; additionally, lectins can interfere with ergosterol biosynthesis and lysosomal stability as well as inhibiting the yeast-to-hyphae transition [31,32,33,34,35,36,37,38,39,40,41,42,43,44,45,46,47,48,49,50,51,52]. For most of the mechanisms described below, the findings are based on correlative evidence, including measurements of reactive oxygen species (ROS) or ATP levels, mitochondrial membrane potential, apoptosis markers, DNA fragmentation or TUNEL assays, microscopy observations, LC/MS proteomics, and gene expression analyses. Direct evidence, by contrast, is limited to studies demonstrating carbohydrate-dependent interactions, such as inhibition by specific sugars or lectin binding detected using fluorescently labeled conjugates.

**Figure 2:**
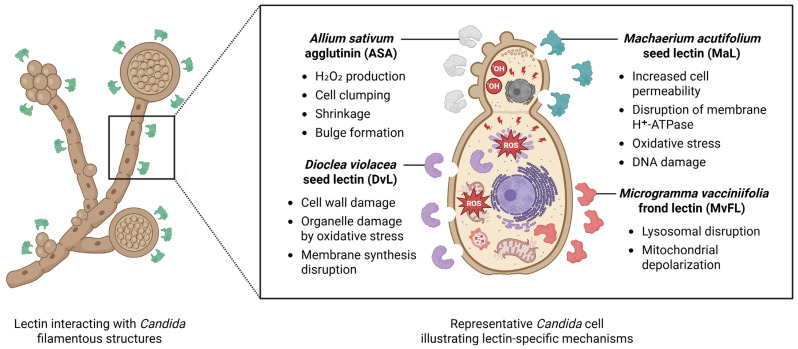
Schematic overview of lectin-specific mechanisms of action on *Candida*. The figure illustrates lectin interactions with filamentous *Candida* structures and a zoomed representative fungal cell used as a schematic model to summarize lectin-specific effects reported in the literature. Distinct mechanisms are highlighted for individual lectins: *Allium sativum* agglutinin (ASA) stimulates H_2_O_2_ production, resulting in cell clumping, shrinkage, and bulge formation; *Dioclea violacea* seed lectin (DvL) induces cell wall damage, interferes with membrane biosynthesis, and promotes organelle impairment; *Machaerium acutifolium* seed lectin (MaL) increases membrane permeability, disrupts H^+^-ATPase function, triggers oxidative stress, and leads to DNA damage; and *Microgramma vacciniifolia* frond lectin (MvFL) targets intracellular compartments, causing lysosomal disruption and mitochondrial depolarization. The illustrated effects represent individual lectin-mediated actions and do not imply simultaneous or synergistic activity within a single fungal cell. Created in BioRender. Lira, T.L.S. (2025) https://BioRender.com/aznf0t7.

The following paragraphs explore the antifungal mechanisms of selected lectins, whose modes of action have been studied in greater depth. In this context, Figure 2 illustrates the mechanisms of some of these anti-*Candida* lectins (DvL, MaL, ASA, and MvFL), highlighting the diverse antifungal mechanisms.

The lectin extracted from the *Dioclea violacea* Mart. ex Benth. seeds (DvL) has demonstrated activity against *C. albicans*, *C. krusei*, and *C. parapsilosis* [38]. The authors reported that DvL caused cell wall damage forming pores ranging from 1.3 to 2.3 µm, depending on the yeast species, induced overproduction of reactive oxygen species (ROS), and inhibited ergosterol biosynthesis (Figure 2). DvL also induced apoptosis, as indicated by cytochrome c release from the mitochondrial membrane.

A gel-free proteomic analysis by liquid chromatography/mass spectrometry (LC/MS) analysis was used to investigate the action of DvL on *C. albicans* and *C. krusei* cells in greater depth. For *C. albicans*, the findings indicate that DvL modulates the expression of proteins associated with cell wall synthesis, oxidative stress response, carbohydrate metabolism, DNA repair, RNA regulation and processing, intracellular protein transport, and the cell cycle [39]. Regarding *C. krusei*, treatment with DvL led to upregulation of proteins associated with cell-wall remodeling and antioxidant responses, while proteins involved in the synthesis of cell-wall precursors, ergosterol metabolism, and detoxification processes were downregulated. Additionally, some proteins were detected exclusively in DvL-treated cells, indicating a metabolic shift toward the glyoxylate cycle, induction of multidrug-efflux proteins, and activation of multiple DNA-repair pathways. [40]. Together, these results demonstrate that DvL exhibits multifaceted antifungal activity against clinically relevant *Candida* species by targeting multiple cellular pathways, which may reduce the likelihood of resistance development. Thus, DvL is as a promising candidate for the development of novel antifungal strategies.

The concanavalins A (ConA) and M (ConM), isolated from *Canavalia ensiformis* (L.) DC. and *Canavalia rosea* (Sw.) DC. seeds, respectively, inhibited the growth of *C. albicans* and *C. tropicalis* as well as were effective in inhibiting yeast-to-hyphae morphological transition in both species [37]. Although the IC_50_ values were high (Table 1), the dual activity of ConA and ConM–targeting both fungal proliferation and a key virulence mechanism–could be valuable in reducing pathogenicity, limiting tissue invasion, and potentially overcoming challenges posed by drug-resistant *Candida* strains.

Q-Griffithsin (Q-GRFT), a recombinant and oxidation-resistant variant of Griffithsin (a lectin derived from marine red algae), exhibited anti-*Candida* activity by binding to α-mannan in *C. albicans*, disrupting cell wall integrity, and inducing ROS formation, which led to cell death. Moreover, Q-GRFT inhibited the growth of *N. glabratus*, *C. parapsilosis*, *C. krusei*, and *C. auris*. It also induced differential expression of numerous genes involved in stress response, ROS neutralization, and cell cycle regulation [50,53].

DvL, ConA, ConM, and GRFT are glucose/mannose-binding lectins. These lectins likely interact with *Candida* because its outer cell wall is rich in exposed mannoproteins and underlying glucose-based β-glucans, providing abundant carbohydrate targets. By binding to and cross-linking these sugars, they can disrupt cell wall organization and impair essential fungal functions. Additionally, GRFT can bind to *N*-acetyl-glucosamine (the monomer of chitin) and α-mannan (a polysaccharide composed of mannose units), further expanding its capacity to target fungal cell walls.

Other lectins able to bind mannose have demonstrated antifungal activity on *Candida* species. The *Helianthus annuus* L. jacalin (Helja) binds to *C. albicans* cell wall mannans, inhibiting growth, reducing viability, inducing morphological changes, and causing cell agglutination [42]. The *Allium sativum* L. agglutinin (ASA) showed fungistatic action and reduced viability of *C. auris* and *N. glabratus* cells, with detection of cellular alterations, including induction of hydrogen peroxide production, cell clumping, shrinkage, loss of cellular integrity and bulge formation (Figure 2) [32]. The lectin from *Machaerium acutifolium* Vogel seeds (MaL) showed inhibitory and fungicidal effects against *C. parapsilosis* and was found to increase cell membrane permeability, disrupt the activity of plasma membrane proton-pumping ATPase, trigger oxidative stress, and induce DNA damage (Figure 2) [43]. In addition to mannose, MaL binds to *N*-acetyl-glucosamine. The *Abelmoschus esculentus* (L.) Moench leaf lectin (AEL) exhibited both fungistatic and fungicidal activity against *C. parapsilosis,* and the use of AEL–FITC conjugates demonstrated strong binding of AEL to *C. parapsilosis* blastoconidia, while only weak binding was observed with *C. tropicalis* and *C. albicans* [31], indicating a species-specific antifungal activity.

Bazán et al. [54] investigated the infectivity of *C. albicans* isolates in *Tenebrio molitor* L. larvae and the protective effects of lectins ConBr and MaL. When lectins were administered before infection, both significantly increased larval survival. After infection, only MaL provided a notable survival benefit, showing that this lectin can act prophylactically and therapeutically.

Some antifungal lectins display chitin-binding properties. The lectin extracted from the juicy sarcotesta of *Punica granatum* L. (PgTeL) exhibited antifungal activity against *C. albicans* and *C. krusei* by compromising cell viability even at sub-inhibitory concentrations. PgTeL treatment induced oxidative stress, decreased ATP levels, and caused structural damage to the yeast cell wall [49]. The water-soluble lectin extracted from *Moringa oleifera* Lam. seeds (WSMoL) demonstrated inhibitory and fungicidal effects on *C. albicans*, *C. krusei*, *C. parapsilosis*, and *N. glabratus*. Exposure to WSMoL led to an increased proportion of yeast cells undergoing apoptosis and necrosis. Additionally, treatment with this lectin resulted in mitochondrial membrane hyperpolarization after 12 h, followed by depolarization at the 24 h mark [52]. In addition to chitin, WSMoL binds to *N*-acetyl glucosamine, glucose and fructose [55]. Other chitin-binding lectins, including those from *Portulaca elatior* Mart. ex Rohrb. leaf (PeLL) and root (PeRoL), as well as *Schinus terebinthifolia* Raddi. leaf (SteLL), also exhibit anti-*Candida* activity [47,48,51].

*Mo*-CBP_2_, another chitin-binding lectin from *M. oleifera* seeds, exhibited antifungal activity against *C. albicans* and caused pronounced cell damage. It was observed surface alterations such as pore-like depressions and collapsed cells, along with consistent leakage of cytoplasmic contents. The antifungal effect was completely abolished by mannose, sucrose, and galactose, suggesting carbohydrate-dependent interactions, and absorbance analyses showed that *Mo*-CBP_2_ also promotes aggregation of *C. albicans* cells [45].

Chitin-binding lectins are able to interact with a key structural component of the fungal cell wall. This interaction can disrupt cell wall integrity, inhibiting growth and hyphal formation, and can trigger stress responses leading to cell death. Additionally, lectin binding may expose fungal components that enhance recognition and clearance by the host immune system. These combined effects make chitin-binding lectins promising antifungal agents against *Candida*.

The lectin derived from *Microgramma vacciniifolia* fronds (MvFL), which binds to oligosaccharides (like those present in fetuin and ovalbumin), demonstrated fungistatic properties against *C. albicans*, *C. krusei*, *C. parapsilosis*, *C. tropicalis*, and *N. glabratus*. In the case of *N. glabratus*, MvFL markedly suppressed cell proliferation, compromised lysosomal membrane stability, and led to a reduction in mitochondrial membrane potential (Figure 2) [44]. The leaf lectin from *Calliandra surinamensis* Benth. (CasuL) demonstrated antifungal activity against *C. krusei*, with treated cells exhibiting pronounced morphological alterations, including cytoplasmic retraction, cell rupture, and accumulation of cellular debris. Furthermore, incomplete budding or division events were observed, and CasuL was found to compromise the structural integrity of the *C. krusei* cell wall. CasuL recognizes oligosaccharide moieties, such as those present in ovalbumin, fetuin and bovine serum albumin [35]. Lastly, the lectin obtained from the inflorescence of *Alpinia purpurata* (Vieill.) K.Schum. (ApuL) exhibited a fungistatic effect on *C. albicans* and *C. parapsilosis*. Treatment with ApuL led to structural alterations in the yeast cells, including malformations, elongation, and bulging [34]. ApuL is also able to interact with oligosaccharides found in glycoproteins [56].

Oligosaccharide-binding lectins can exert antifungal activity primarily by interacting with glycans present on *Candida* cell walls, particularly those attached to cell wall glycoproteins. These glycoproteins are essential for maintaining structural integrity, mediating adhesion, and supporting wall remodeling during cell growth and division. Lectin binding to these glycoproteins can disrupt the organization of the cell wall matrix, leading to morphological changes such as cytoplasmic retraction, bulging, and cell rupture.

In summary, the anti-*Candida* efficacy of lectins is tightly governed by their carbohydrate-binding specificity, with each sugar target—mannose, glucose, chitin, or complex oligosaccharides—dictating the spectrum and mechanism of activity. Lectins that recognize abundant or structurally critical cell wall components, such as mannans or chitin, tend to induce pronounced fungicidal effects through cell wall disruption and oxidative stress.

## 4. Antifungal Activity of Lectins on *Cryptococcus* Species

**Figure 3:**
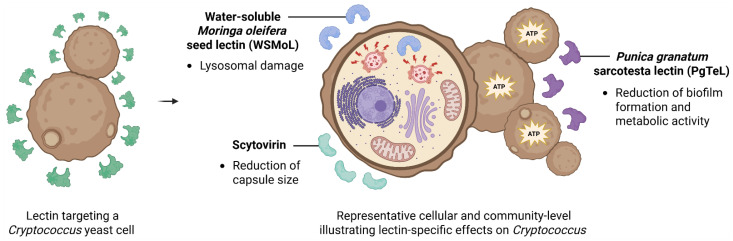
Conceptual models illustrating lectin-mediated mechanisms of action on *Cryptococcus*. The left panel illustrates lectin interactions with *Cryptococcus* yeast cells. The mechanistic panel presents representative cellular and community-level models summarizing distinct effects of individual lectins described in the literature. WSMoL induces lysosomal damage, affecting intracellular compartment integrity. Scytovirin is associated with a reduction in capsule size, impacting capsular architecture. PgTeL reduces biofilm formation and metabolic activity, indicating impaired cellular function at the community level. The depicted effects represent independent lectin-mediated mechanisms and do not indicate simultaneous or synergistic actions within a single cell or biofilm. Created in BioRender. Lira, T.L.S. (2025) https://BioRender.com/ugxjnac.

Despite advances in the study of natural products with antifungal activity, most research has focused on yeasts of the *Candida* genus, while studies involving *Cryptococcus* species remain scarce. This gap concerns the clinical relevance of these species, especially in immunocompromised patients. Furthermore, the therapeutic potential of natural compounds, such as lectins, against *Cryptococcus* is still largely underexplored. This points to the need for further research aimed at developing innovative and effective therapeutic alternatives in response to the growing resistance to conventional antifungal agents. Nevertheless, some lectins have already been investigated for their anticryptococcal activity (Table 2). Figure 3 summarizes the effects of three of these anti-*Cryptococcus* lectins, scytovirin, PgTeL and WSMoL.

The cyanobacterial lectin Scytovirin has demonstrated fungicidal activity against strains of *C. neoformans* (serotypes A and D) and *C. gattii* as well as affected capsule size and prevented the release of the polysaccharide capsule (Figure 3) [60]. Scytovirin specifically binds high-mannose oligosaccharides, a property that may underline its interaction with mannan polymers and mannoproteins present in both the capsule and the cell membrane of *Cryptococcus* species. These interactions likely contribute to the observed disruptions in capsule integrity and fungal viability.

WSMoL showed fungistatic effect against *C. neoformans* strains H99 and B3501, and *C. gattii* strain R265. This lectin promoted lysosomal damage in B3501 and R265 cells and reduced the mitochondrial membrane potential in B3501 (Figure 3) [61]. The *Myracrodruon urundeuva* Allem. heartwood lectin (MuHL) exhibited fungistatic and fungicidal activities against *C. neoformans* H99 and B3501 and *C. gattii* R265 [58] while PgTeL inhibited the growth of *C. neoformans* B3501 [59]. The anti-*Cryptococcus* activities observed for WSMoL, MuHL, and PgTeL can be interpreted in light of their shared classification as chitin-binding lectins and the structural and functional characteristics of the *Cryptococcus* cell wall. Chitin, although less abundant than glucans, is an essential component of the cryptococcal cell wall and is particularly enriched at sites of cell division and wall remodeling. The differential susceptibility among strains may reflect variations in cell wall architecture, chitin exposure, or compensatory stress response pathways.

The cMoL, a galactose-binding lectin from *M. oleifera seeds*, exhibited fungistatic activity against the strains H99, B3501, and R265, inducing necrosis and apoptosis in fungal cells [57]. Unlike the chitin-binding lectins discussed above, cMoL recognizes galactose-containing glycoconjugates. These are also present in cryptococcal surface structures, including cell wall polysaccharides and capsule-associated glycans like galactoxylomannan.

## 5. Antibiofilm Activity of Lectins on Yeasts

The antibiofilm activity of lectins against *Candida* species has also been investigated. Lectins such as ApuL, PgTeL, and a lectin isolated from *Triticum aestivum* L. seeds were shown to inhibit *C. albicans* biofilm formation [34,49,62], whereas ASA exhibited antibiofilm activity against *C. auris* and *N. glabrata* [32]. In contrast, MvFL displayed minimal antibiofilm activity, causing only a slight reduction in biofilm formation by *C. tropicalis* [44]. *Mo*-CBP_2_ inhibited biomass formation by *C. albicans* and *C. tropicalis* during the initial adhesion stage. In addition, *Mo*-CBP_2_ reduced the biomass of mature biofilms of both species by approximately 50–75%, although this effect was not dose dependent [46]. Despite these promising findings, the molecular mechanisms underlying the antibiofilm activity of lectins against *Candida* remain unexplored.

With respect to antibiofilm activity of lectins on *Cryptococcus*, PgTeL reduced biofilm biomass of *C. neoformans* B3501 over a wide concentration range (4–256 µg/mL) and decreased metabolic activity of biofilm-embedded cells at concentrations ≥32 µg/mL. Moreover, PgTeL (8–256 µg/mL) was also able to eradicate pre-formed biofilms of this strain [59]. These results indicate PgTeL activity not only during the initial stages of biofilm development but also against established biofilm structures (Figure 3). However, the molecular basis of these antibiofilm effects remains to be elucidated.

WSMoL (25–400 µg/mL) was also able to inhibit biofilm formation by the B3501 strain [61], reinforcing the relevance of chitin-targeting strategies in interfering with cryptococcal biofilms. On the other hand, cMoL did not demonstrate antibiofilm activity [57], suggesting that galactosylated targets recognized by this lectin are either less accessible or less critical for biofilm establishment and maintenance in *C. neoformans*.

## 6. Combination Effects of Lectins with Antifungal Drugs

Evaluating the synergistic interactions between antifungal agents and lectins is essential for improving treatment outcomes, as it may enhance antifungal efficacy, reduce required drug dosages, minimize side effects, and help overcome the increasing challenge of antifungal resistance. To assess drug interactions, fractional inhibitory concentrations (FICs) and the fractional inhibitory concentration index (FICI) are calculated. The FIC for each agent is defined as the MIC in combination divided by the MIC alone. The FICI is then obtained by summing the FICs of the antifungal drug and the lectin. Interactions are interpreted as follows: FICI ≤ 0.5, synergism; 0.5 < FICI ≤ 1, additive effect; 1 < FICI ≤ 2, no interaction (indifferent); and FICI > 2, antagonism.

**Figure 4:**
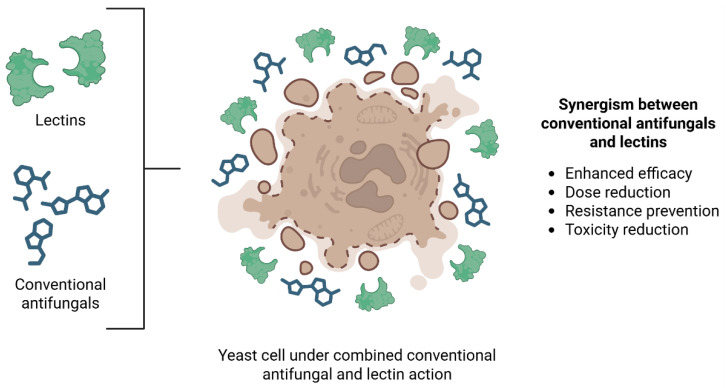
Synergistic antifungal activity between lectins and conventional antifungals. The combined action of lectins and conventional antifungal agents enhances fungicidal efficacy by simultaneously targeting membrane and intracellular structures, resulting in extensive cell damage and apoptosis-like cell death. This cooperative mechanism improves therapeutic efficiency while contributing to lower effective doses, reduced adverse effects, and decreased likelihood of resistance development. Created in BioRender. Lira, T.L.S. (2025) https://BioRender.com/gb481k9.

Indeed, lectins have also been shown to enhance the efficacy of conventional antifungal agents through synergistic interactions against yeast species (Figure 4). Table 3 summarizes data on investigation of combinations between lectins and conventional antifungal drugs against *Candida* and *Cryptococcus*.

Helja significantly increased the antifungal activity of fluconazole against *C. albicans*. In addition, treated cells displayed pronounced morphological abnormalities, including nuclear disintegration and multimeric structure formation, which ultimately led to cellular collapse [41]. ApuL demonstrated synergistic antifungal activity in combination with fluconazole against *C. parapsilosis* [34] and ConA and ConM showed additive effect when combined with fluconazole at subinhibitory concentrations, enhancing the antifungal effectiveness against *C. albicans* by over 50% [37]. Such synergistic or additive interactions can be therapeutically advantageous, allowing lower doses of each agent, potentially reducing toxicity and limiting the development of drug resistance. Synergism may arise from complementary mechanisms of action, although the exact molecular basis often requires further investigation.

In the case of MvFL, combination studies revealed synergistic interaction with fluconazole when assessed against *C. parapsilosis*. However, antagonistic effects were observed for the combination MvFL with fluconazole against *C. albicans* and *N. glabratus* [44], highlighting the importance of species-specific responses when evaluating combination therapies.

Although the antifungal mechanisms of lectins against *Cryptococcus* species are still not fully understood, they showed promising potential as alternative or adjunctive therapies against these pathogenic yeasts. Scytovirin exhibited strong synergistic effects when combined with the commercial antifungal amphotericin B [60]. cMoL showed synergistic effect in combination with fluconazole against the H99 strain of *C. neoformans*. However, the combination proved to be antagonistic for *C. neoformans* B3501 and *C. gattii* R265 [57]. Such antagonistic interactions can be detrimental in clinical settings, potentially reducing therapeutic efficacy and promoting persistence or resistance. The occurrence of antagonism emphasizes the necessity of carefully evaluating the species- and strain-specific responses before proposing combination therapies.

## 7. Translational Challenges

**Figure 5:**
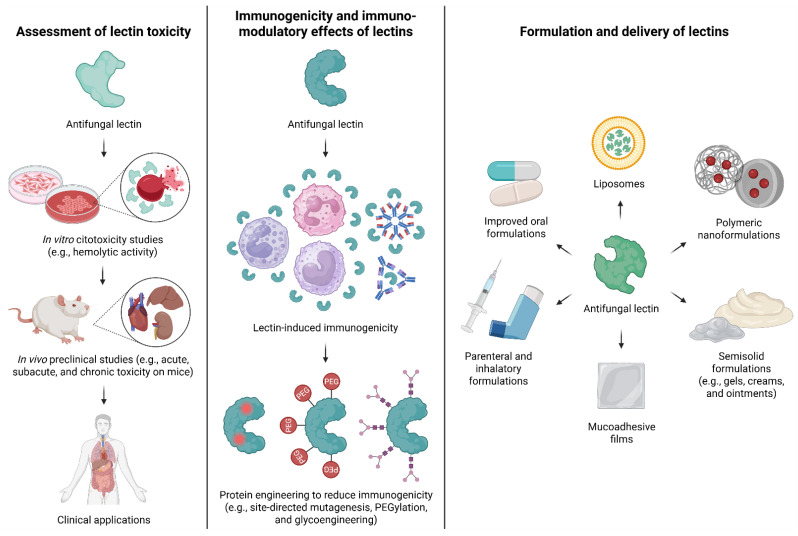
Overview of translational challenges associated with antifungal lectins. The figure summarizes the major translational barriers limiting the clinical application of antifungal lectins, organized into three interconnected axes: toxicity assessment, immunogenicity and immunomodulatory effects, and formulation and drug delivery. The toxicological axis depicts a stepwise pipeline, starting with in vitro cytotoxicity assays (e.g., hemolysis), following to in vivo preclinical studies in animal models evaluating acute, subacute, and chronic toxicity, which collectively support the feasibility of future clinical applications. The immunogenicity axis highlights the potential recognition of lectins by components of the immune system, such as immune cells and antibodies, emphasizing the need to control or reduce immunogenic responses during therapeutic development through protein engineering-based strategies. Finally, the drug delivery axis presents formulation strategies designed to overcome pharmacological limitations of lectins, including improved oral forms, liposomal and polymeric nanoformulations, semi-solid formulations, mucoadhesive films, and parenteral or inhalable delivery systems. Together, these three axes highlight the multidisciplinary challenges that must be integrated to enable the translational development of lectin-based antifungal therapies. Created in BioRender. Lira, T.L.S. (2025) https://BioRender.com/wgpc67n.

As shown above, research on the antifungal activity of lectins against pathogenic yeasts has advanced in recent years, particularly in efforts to elucidate the mode of action of these proteins in different isolates or species. However, in addition to these scientific challenges, several translational bottlenecks must also be addressed, including toxicity, immunogenicity, stability, and delivery strategies (Figure 5). In the following sections, information regarding these aspects for antifungal lectins is compiled, or the absence of important translational considerations is highlighted.

### 7.1. Assessment of Lectin Toxicity

In vitro cytotoxicity studies are crucial for assessing the safety of lectins in biomedical applications, providing a controlled setting to detect potential toxic effects and establish safe concentration ranges. These studies commonly employ non-target cells, including hepatic, renal, cardiac, endothelial, fibroblast, erythrocytes, and immune cells, to evaluate general cytotoxicity before progressing to in vivo experiments. Some antifungal lectins listed in Table 1 and Table 2 have been investigated in this context. CasuL, for instance, did not reduce the viability of human peripheral blood mononuclear cells at concentrations below 100 μg/mL and, across 3.12–100 μg/mL, failed to induce apoptosis or necrosis in mouse splenocytes [35,63]. Similarly, SteLL was non-toxic to splenocytes and did not affect cytosolic Ca^2+^ levels or reactive oxygen species production [64], while MaL did not cause reduction in RAW 264.7 macrophage viability at 31.25 μg/mL [65].

Hemolytic activity does not appear be a limitation for lectins such as PeLL, which caused a maximum of 14.3% hemolysis in human erythrocytes at 300 μg/mL [47], and PgTeL, which exhibited no cytotoxicity to murine splenocytes within 1.56–50 μg/mL and no hemolysis up to 1000 μg/mL [66]. In contrast, DvL demonstrated measurable cytotoxicity toward Vero (monkey kidney) and HaCaT (human fibroblast) cells, with IC_50_ values of 71.6 and 80.2 μg/mL, corresponding to 2.8 and 3.1 µM, respectively [67]. Notably, ASL50 showed no cytotoxic effects on HEK 293 (embryonic kidney) cells or human erythrocytes [33].

Together, these studies illustrate the diverse safety profiles of antifungal lectins and highlight the importance of systematic cytotoxicity screening. Establishing non-toxic concentration ranges in vitro not only informs safer application strategies but also lays the groundwork for preclinical in vivo studies, which have already been conducted for some of the antifungal lectins reviewed in this paper.

PgTeL, for example, was evaluated for acute toxicity in Swiss mice. At a dose of 100 mg/kg (i.p.), no differences were observed in food or water intake, nor in body weight variation, and the compound did not induce any behavioral signs of toxicity. Treated animals also exhibited no alterations in their coagulogram (activated partial thromboplastin time, prothrombin time, and thrombin time), hematological parameters, or blood biochemical parameters, except for reductions in triglyceride, LDL, and VLDL levels. Additionally, the relative organ weights remained unchanged. PgTeL was also non-genotoxic in vivo at the same dose of 100 mg/kg (i.p.) [66].

A single dose of SteLL (100 mg/kg), administered orally or intraperitoneally in Swiss mice, caused no mortality or signs of toxicity over a 14-day period. Water and food intake, body weight, hematological and biochemical parameters, and organ weights were not significantly affected, except for a slight increase in food intake following intraperitoneal administration. Histopathological analysis revealed normal architecture of the liver, kidney, spleen, and stomach [68].

The in vivo acute toxicity of PeLL was also assessed in Swiss mice following oral administration at doses of 5 and 10 mg/kg. Over the 14-day observation period, no significant changes were observed in body weight, water intake, or food consumption compared to the control group. Hematological parameters remained within normal ranges, and most biochemical markers were unaffected, although mice treated with 10 mg/kg PeLL exhibited a slight decrease in serum albumin levels [47].

Acute toxicity of cMoL was assessed in Swiss mice following a single dose of 200 mg/kg administered orally or intraperitoneally. Genotoxic potential was evaluated using the comet assay and micronucleus test. At this dose, cMoL did not cause mortality or observable toxic effects, and no alterations were detected in hematological, biochemical, histopathological, or genotoxic parameters [57].

GRFT exhibited minimal toxicity in rodents following single or repeated subcutaneous doses. In BALB/c mice, a single 50 mg/kg dose or 14 daily doses of 10 mg/kg were well tolerated, with no significant changes in general health, blood chemistry, or complete blood counts. In Hartley guinea pigs, mild and reversible increases in liver and spleen mass were observed, but no histopathological alterations occurred. Across subcutaneous, intravaginal, and intraperitoneal administrations relevant to microbicide development, GRFT demonstrated a safety profile, with reversible splenomegaly and activation of certain spleen B and T cells, but no adverse effects on other organs [69,70].

These results show that the lectins PgTeL, SteLL, PeLL, cMoL, and GRFT were generally safe in rodent studies, causing no significant toxic effects. Some changes observed were mild and reversible, supporting further studies on their translational relevance for future preclinical research as antifungal drug candidates. However, additional administration routes, such as dermal, intradermal, intranasal, and mucosal, should be evaluated, and studies with other doses and longer observation periods are needed to better define their safety profile.

In contrast, some of the other lectins mentioned in the review have been reported as toxic in rodents depending on the dose. WSMoL exhibited a lower safe profile in mice compared to cMoL since its intraperitoneal administration at doses of 100 mg/kg or higher proved hazardous. Animals receiving WSMoL at 200 mg/kg i.p. exhibited depressive behaviors, lethargy, constipation, and abdominal spasms within the first hour after administration. At this dose, mortality reached 40% after three days. Conversely, when the dose was reduced to 100 mg/kg i.p., no deaths were observed; however, animals displayed mild abdominal contractions, decreased mobility, tail erythema, lethargy, prostration, and constipation [71]. ConA also requires careful handling, as it effectively induces liver inflammation, with toxicity varying according to the dose. Intravenous administration triggers liver injury by activating and recruiting T cells. This effect could begin at 15 mg/kg i.v., depending on the mouse strain [72]. In the case of DVL, animals were injected subcutaneously into the left hind footpad with 50 µg of lectin and developed lymph nodes exhibiting apoptotic foci and focal inflammatory reactions, characterized by abundant macrophages and neutrophils, as well as hemorrhage [73]. These findings emphasize the importance of guiding safe dosing in future preclinical studies.

MvFL (10 and 20 mg/kg, i.p.) reduced sarcoma 180 tumor growth in mice by inducing necrosis and leukocyte infiltration, as well as by interfering with angiogenesis [74]. The authors also evaluated the animals for toxicity and observed no changes in water or food consumption, body weight, hematological or biochemical parameters, and no signs of toxicity in the liver, kidneys, or spleen.

Other lectins have been evaluated pharmacologically in vivo, although systematic evaluations of their acute, subacute, or chronic toxicity were not the focus. For instance, AEL exhibited anti-inflammatory effects in a paw edema model in Wistar rats when administered intravenously at doses of 0.01, 0.1, and 1 mg/kg [75]. The topical application of BVL (200 μg/mL) on dorsal skin wounds in Swiss albino mice promoted healing, indicating a potential role in tissue repair [76]. ConBr has shown neuroprotective activity against quinolinic acid-induced seizures following intracerebroventricular administration at 10 μg per site [77]. Additionally, ConBr (1–10 μg/site, i.c.v.) reduced immobility time in the forced swim test without altering locomotor activity in the open-field test, suggesting potential antidepressant-like effects [78]. In a model of acute pancreatitis, intravenous administration of ConBr (0.1, 1, or 10 mg/kg) at 1 and 12 h post-induction mitigated pancreatic damage, including neutrophil infiltration, edema, and necrosis [79]. Although these studies demonstrate the therapeutic potential of lectins, comprehensive toxicity data are still lacking. Detailed acute, subacute, and chronic toxicity evaluations are essential to fully assess their safety and support the translational development of lectins for clinical applications, including their antifungal properties highlighted in this review.

### 7.2. Immunogenicity and Immunomodulatory Effects of Lectins

The assessment of protein immunogenicity is particularly critical for pharmaceutical applications, such as oral or mucosal delivery, since immune recognition of a lectin could interfere with its therapeutic efficacy. Due to their proteinaceous nature, lectins can be immunogenic because their amino acid sequences, three-dimensional structures, or unique post-translational modifications may be recognized as foreign by the animal immune system. This recognition can stimulate the production of antibodies or activate immune cells, potentially leading to allergies or inflammatory responses.

However, evaluating lectins is especially complex because some of these proteins are immunomodulatory and can enhance immune responses for a desirable purpose. As examples of antifungal lectins with immunomodulatory activity, CasuL exhibited effects on mice splenocytes: treatment stimulated splenocyte proliferation, increased cytosolic ROS levels, did not affect cytosolic calcium concentration, mitochondrial ROS, or ΔΨm levels, and promoted the release of IL-2 and TNF-α [63]. SteLL induced splenocytes to release pro-inflammatory cytokines (IL-17A, TNF-α, IFN-γ, and IL-2) as well as IL-4, an anti-inflammatory cytokine that can prevent excessive inflammation [64]. MvFL enhanced lysosomal activity in murine macrophages, which may have contributed to its leishmanicidal effect, although it did not alter the phagocytic capacity of macrophages [80]. MvFL (5 and 10 mg/kg, i.p.) also exhibited in vivo anti-inflammatory activity in peritonitis and paw edema models by modulating leukocyte infiltration and cytokine release [81].

DVL exerts a proliferative effect on RAW 267.4 macrophages [82]. However, its immunological effects should be considered with caution. As noted in the previous subsection, DVL administration stimulated the lymph nodes of BALB/c mice in a manner that induced apoptosis of leukocytes in the parafollicular, interfollicular, and subcapsular regions, with few affected cells in the medullary areas. This previously reported pro-inflammatory effect of DVL is concerning, as it led to hemorrhagic lymph nodes accompanied by fibrinoid necrosis [73]. It was also mentioned above that ConA and GRFT can modulate the immune response in rodents, which, as observed, can be either positive or negative depending on the context and dose.

Studies in animal models have demonstrated the immunogenicity of lectins, particularly their ability to induce antibody production. For example, mistletoe (*Viscum album* L.) lectin 1 induces strong systemic IgG and IgA responses, as well as mucosal IgA, whereas lectins such as PHA (*Phaseolus vulgaris* L. agglutinin), WGA (*T. aestivum* agglutinin), and UEA-1 (*Ulex europaeus* L. agglutinin) generate relatively weak antibody responses with minimal mucosal involvement. These differences evidence the importance of evaluating each lectin individually rather than assuming uniform effects [83,84,85].

Regarding the antifungal lectins discussed in this review, the immunogenic potential of garlic lectins (ASA) following oral administration in BALB/c mice was evaluated, revealing a lectin-specific serum IgG response. The immunogenicity of ASA was associated with its moderate stability in simulated gastric fluid [86]. Furthermore, intradermal and intranasal administration of ASA I and ASA II in BALB/c mice led to marked increases in anti-lectin IgG levels, without affecting body weight, while also inducing enlargement of the spleen and thymus. Notably, intranasal co-administration of ASA I with ovalbumin (OVA) significantly enhanced anti-OVA IgG responses, highlighting its strong capacity to elicit mucosal immunity [87].

To minimize immunogenicity and undesired immunomodulatory effects of lectins, a combination of strategies can be employed: protein engineering (e.g., site-directed mutagenesis, PEGylation, or glycoengineering) can mask immunogenic epitopes; controlled and targeted delivery using nanoparticles, liposomes, or mucosal carriers can limit systemic immune exposure; careful dosing regimens, including gradual escalation or co-administration with tolerogenic agents, can reduce antibody responses; selection of naturally low-immunogenic lectins and avoidance of strongly pro-inflammatory ones helps prevent adverse effects.

### 7.3. Formulation and Delivery of Lectins

In continuity, it is evident that, despite their promising therapeutic potential, the clinical application of antifungal lectins still faces fundamental challenges related to their safe and effective use as drugs. In this context, protective strategies and drug delivery systems can help mitigate undesired toxicity and immunogenicity, while also enhancing bioavailability and enabling targeted delivery. The treatment of yeast infections depends on their severity: superficial infections, such as mild cutaneous or vaginal candidiasis, are usually treated with topical antifungals, while severe, recurrent, or systemic cases may require oral therapy.

Delivering lectins orally remains difficult, similar to what is observed with other proteins, because of their physicochemical characteristic as well as numerous physiological barriers throughout the gastrointestinal tract. These macromolecules are exposed first to the highly acidic gastric environment, where proteolytic enzymes like pepsin and cathepsin can rapidly degrade them. Fluctuations in stomach pH can further destabilize their conformation and impair their function. Degradation continues in the small intestine via trypsin, α-chymotrypsin, and other enzymes, leading to extremely low bioavailability, often below 1% [88,89]. To address these challenges, a variety of strategies have been explored to enhance the stability, permeability, and overall performance of orally delivered protein therapeutics.

One strategy involves chemically modifying lectins. Lipidation increases lipophilicity, improving half-life, reducing immunogenicity, and enabling intracellular and epithelial delivery, though it may affect structure or receptor binding [89,90,91]. PEGylation shields antigenic sites, reduces degradation, and enhances stability and pharmacokinetics, but can increase size, reduce cellular affinity, and raise biocompatibility concerns [88,92]. Other modifications, like esterification and cationization, improve lipophilicity or membrane interaction, respectively, but may increase plasma protein binding, immunogenicity, or clearance [88,89].

Reducing enzymatic breakdown is another strategy. Protease inhibitors protect protein drugs from enzymatic degradation but may cause toxicity or disrupt pancreatic function, while mucolytics enhance epithelial drug access by reducing mucus viscosity, though excessive thinning can increase acid and enzymatic vulnerability of the protein [88,89].

Various delivery systems have been developed to protect proteins and enhance their therapeutic efficacy, including microemulsions [93], self-emulsifying systems [94], liposomes [95], and polymeric nanoparticles [96]. These systems shield proteins from enzymatic degradation, enable controlled release, improve stability, and facilitate intestinal absorption. However, microparticles face challenges like limited loading, aggregation, and scale-up issues, while liposomes are constrained by stability, leakage, and short shelf life [88,89,97]. Considering the antifungal lectins reviewed here, liposomes loaded with BVL enhance cellular uptake and cytotoxicity while stabilizing protein structure [98]. DVL in controlled-release CaCO_3_ particles also showed efficient lectin release and higher cytotoxicity against HeLa cells compared to free lectin [99]. However, studies using lectins in delivery systems for anti-*Candida* and anti-*Cryptococcus* applications are still needed.

Since each strategy has distinct advantages and limitations, combining complementary approaches represents the most promising path toward clinically reliable protein-based oral therapies. While oral administration remains challenging, lectins with antifungal activity are likely better suited for topical delivery, where protein stability is maintained and localized action is possible. These lectins could potentially be formulated into gels, creams, ointments, or mucoadhesive films for cutaneous, oral, or vaginal candidiasis; into vaginal mucoadhesive gels for prolonged residence; into inhalable forms such as nebulized suspensions or dry powders for pulmonary *Cryptococcus* infections; and into parenteral formulations, including injectable solutions or lipid/polymeric nanoparticles, for systemic infections. These strategies aim to maintain stability and efficacy, though some remain theoretical and have not yet been experimentally tested.

Vaginal treatment of CBA/J mice with a 1% Q-GRFT prototype formulated in Carbopol gel resulted in a reduced *C. albicans* burden without altering the numbers of vaginal neutrophils or monocytes. Histopathological analysis also showed decreased vaginal colonization by *C. albicans* following Q-GRFT treatment [53]. Thus, Q-GRFT represents a promising broad-spectrum antifungal candidate with potential for topical application.

Alginate-based films incorporating MaL or ConBr lectins have been developed and evaluated ex vivo for mucoadhesive properties using bovine jugal mucosa. Both MaL- and ConBr-loaded films were biocompatible and structurally stable, releasing lectins at similar initial rates, though MaL reached a higher final release. MaL films exhibited smoother surfaces, whereas ConBr films showed greater mucoadhesiveness. Despite these differences, neither lectin altered core film properties such as thickness, moisture, pH, or swelling, and both formulations dissolved rapidly, demonstrating their suitability as orodispersible delivery systems [54]. However, antifungal activity against *C. albicans* was not assessed in the work.

In another approach, an antimicrobial material was designed for wound dressings or coatings by immobilizing *Pseudomonas aeruginosa* lectin B on a protein-based hydrogel, enabling the material to capture pathogens on contact. LecB mediated strong binding of laboratory and clinical *P. aeruginosa strains*, and the drug-loaded fibrillar compartment subsequently eliminated the captured bacteria. This integrated strategy has potential to improve management of extensive hospital wounds by preventing biofilm formation and controlling aggressive, carbapenem-resistant strains [100], and thus it could be adapted for antifungal lectins in the future.

### 7.4. Knowledge Gaps and Future Directions

Despite growing evidence that various lectins exhibit significant antifungal activity against pathogenic yeasts, their translational development as therapeutic agents remains largely unexplored. Most studies to date have focused on in vitro antifungal efficacy, while comprehensive investigations into pharmacological development are scarce or limited, particularly formulation, delivery strategies, stability, bioavailability, and toxicity. This represents a critical bottleneck since without optimized delivery systems, thorough pharmacokinetic characterization, and toxicity assessment, even lectins with potent anti-*Candida* and anti-*Cryptococcus* activity cannot progress reliably toward clinical applications.

While some preliminary delivery strategies, such as encapsulation in nanoparticles, liposomes, or mucoadhesive films, have shown promise in protecting lectins and enhancing efficacy, these approaches remain sporadic and underdeveloped when considering antifungal application. For most antifungal lectins, systematic studies integrating chemical modifications, controlled-release carriers, or targeted delivery strategies have not yet been conducted.

These knowledge gaps highlight the urgent need for coordinated research bridging in vitro activity with practical pharmacological applications. Developing formulations that stabilize lectins, enable controlled release, and target specific tissues or infection sites is essential to unlock their therapeutic potential. Furthermore, investigating multiple administration routes (oral, topical, mucosal, and other) alongside rigorous toxicity studies will provide crucial insights into the safety, efficacy, and optimal strategies for effective antifungal therapy.

## 8. Conclusions

The emergence of alternative therapies for fungal infections has become increasingly important in global health. Lectins have demonstrated versatility and significant potential as therapeutic agents against human fungal pathogens, particularly species responsible for infections with high morbidity and mortality, such as those in the genera *Candida*, *Nakaseomyces* and *Cryptococcus*. These molecules act through diverse mechanisms, including disruption of fungal cell membranes, induction of oxidative stress, inhibition of ergosterol biosynthesis, and interference with mitochondrial and lysosomal functions. Some lectins have been shown to inhibit yeast-to-hyphae morphological transitions and biofilm formation, which are critical virulence factors for pathogenic yeasts. Moreover, lectins have shown potential to enhance the efficacy of conventional antifungal drugs through synergistic interactions, suggesting their value as adjunctive therapeutic agents.

Despite these promising findings, the current evidence is uneven: while some studies provide robust mechanistic insights, others remain preliminary, often reporting effects similar to those observed in different cell types or with other molecules. To translate lectins into viable antifungal agents, future research must address several critical gaps: limited investigation of *Cryptococcus* compared to *Candida*; detailed elucidation of molecular pathways mediating antifungal and antibiofilm activities; characterization of synergistic interactions across diverse clinical isolates; in vivo and clinical validation; and assessment of toxicity and delivery challenges. Additionally, potential antagonistic interactions between lectins and certain antifungal drugs must be considered.

At present, promising antifungal lectins remain largely experimental, restricting their contribution to the urgent need for new antifungal agents amid rising drug resistance. Therefore, advancing antifungal lectins toward clinical application requires a concerted effort in rational formulation design, innovative delivery strategies, and rigorous preclinical evaluation to ensure their safe, effective, and practical use as antifungal therapeutics.

## Figures and Tables

**Table 1:** Representative lectins with activity against *Candida* spp. and *Nakaseomyces glabratus*: carbohydrate specificity, experimental data, and observed effects.

Lectin	Binding Carbohydrates	Protocol	Target Fungi	Endpoint (Incubation Time)	Effects	Reference
*Abelmoschus esculentus* leaf lectin (AEL)	Lactose, fructose, mannose	Broth microdilution (CLSI M27-A2)	*C. parapsilosis* FAMED-FURG	MIC: 0.97 µg/mL (48 h) MFC: 31.25 µg/mL (48 h)	Fungistatic Fungicidal	[31]
*Allium sativum* agglutinin (ASA)	Mannose, oligosaccharides	Broth microdilution (CLSI M100)	*C. auris* NCCPF470153	MIC: 42 µg/mL (24 h)	Fungistatic Oxidative stress Morphological alterations Cell wall disruption	[32]
			*C. auris* NCCPF470197	MIC: 69 µg/mL (24 h)		
			*C. auris* NCCPF470200	MIC: 30 µg/mL (24 h)		
			*N. glabratus* NCCPF100037	MIC: 43 µg/mL (24 h)		
			*N. glabratus* NCCPF100033	MIC: 56 µg/mL (24 h)		
			*N. glabratus* ATCC2001	MIC: 33 µg/mL (24 h)		
			*N. glabratus* MTCC3019	MIC: 49 µg/mL (24 h)		
*Allium sativum* bulb lectin 50 (ASL50)	Mannose	Broth microdilution (CLSI M27-A3)	*C. krusei* ATCC6258	MIC: 40 µg/mL (24 h)	Fungistatic Cell wall disruption	[33]
			*C. parapsilosis* ATCC22019	MIC: 10 µg/mL (24 h)		
			*C. tropicalis* ATCC13803	MIC: 20 µg/mL (24 h)		
			*N. glabratus* ATCC15126	MIC: 80 µg/mL (24 h)		
			Clinical isolates	MIC: 10–40 µg/mL (24 h)		
*Alpinia purpurata* inflorescence lectin (ApuL)	Oligosaccharide chains	Broth microdilution	*C. albicans* URM 5901	MIC: 200 µg/mL (24 h)	Fungistatic Morphological alterations	[34]
			*C. parapsilosis* URM 6345	MIC: 400 µg/mL (24 h)		
*Bauhinia variegata* seed lectin (BVL)	Galactose, *N*-acetyl-galactosamine	Broth microdilution (CLSI M27-A2)	*C. parapsilosis* FAMED-FURG	MIC: 125 µg/mL (48 h) MFC: 500 µg/mL (48 h)	Fungistatic Fungicidal	[31]
*Calliandra surinamensis* leaf pinnulae lectin (CasuL)	Oligosaccharide chains	Broth microdilution	*C. krusei* URM 6391	MIC: 125 μg/mL (24 h) MFC: 250 μg/mL (24 h)	Fungistatic Fungicidal Cell wall disruption	[35]
*Canavalia brasiliensis* seed lectin (ConBr)	Glucose, mannose	Broth microdilution (CLSI M27-A2)	*C. parapsilosis* FAMED-FURG	MIC: 125 µg/mL (48 h) MFC: 500 µg/mL (48 h)	Fungistatic Fungicidal	[31,36]
			*C. albicans* URM 4987	MIC: 8 µg/mL (48 h)		
			*C. guilliermondii* URM 4975	MIC: 8 µg/mL (48 h)		
			*C. membranaefaciens* URM 4983	MIC: 2 µg/mL (48 h)		
			*C. shehatae* URM 4978	MIC: 2 µg/mL (48 h)		
			*C. tropicalis* URM 4989	MIC: 8 µg/mL (48 h)		
*Canavalia ensiformis* agglutinin (ConA)	Glucose, Mannose	Broth microdilution	*C. albicans* ATCC 10231	IC_50_: 539.1 µg/mL (24 h)	Fungistatic Inhibition of yeast-to-hyphae transition	[37]
			*C. tropicalis* ATCC 13803	IC_50_: 502.5 µg/mL (24 h)		
*Canavalia rosea* seed lectin (ConM)	Trehalose, Maltose, Glucose, Fructose, Sacarose, Mannose	Broth microdilution	*C. albicans* ATCC 10231	IC_50_: 607.2 µg/mL (24 h)	Fungistatic Inhibition of yeast-to-hyphae transition	[37]
			*C. tropicalis* ATCC 13803	IC_50_: 405.5 µg/mL (24 h)		
*Clitoria fairchildiana* seed lectin	N.I.	Broth microdilution (CLSI M27-A2)	*C. parapsilosis* FAMED-FURG	MIC: 1.95 µg/mL (48 h) MFC: 3.90 µg/mL (48 h)	Fungistatic Fungicidal	[31]
*Dioclea rostrata* seed lectin (DRL)	Glucose, Mannose	Broth microdilution (CLSI M27-A2)	*C. guilliermondii* URM4975	MIC: 128 µg/mL (48 h)	Fungistatic	[36]
			*C. membranaefaciens* URM4983	MIC: 64 µg/mL (48 h)		
			*C. shehatae* URM4978	MIC: 4 µg/mL (48 h)		
*Dioclea virgata* seeds lectin	Complex sugars	Broth microdilution (CLSI M27-A2)	*C. parapsilosis* FAMED-FURG	MIC: 3.9 µg/mL (48 h)	Fungistatic	[31]
*Dioclea violacea* seed lectin (DvL) ^a^	Glucose, Mannose	Broth microdilution (CLSI M27-A3)	*C. albicans* ATCC 10231	MIC: 0.6 µM (24 h)	Fungistatic effect Cell wall damage Oxidative stress Inhibition of ergosterol biosynthesis Apoptosis	[38,39,40]
			*C. krusei* ATCC 6258	MIC: 9.8 µM (24 h)		
			*C. parapsilosis* ATCC 22019	MIC: 0.6 µM (24 h)		
*Helianthus annuus* jacalin (Helja)	Mannose	Broth microdilution	*C. albicans* NGY152	MIC: 65 µg/mL (30 h)	Morphological alterations Binding to mannans	[41,42]
*Machaerium acutifolium* seed lectin (MaL) ^a^	Mannose, *N*-acetyl-glucosamine	Broth microdilution	*C. parapsilosis* ATCC 22019	MIC/MFC: 18 µM (24 h)	Fungistatic Fungicidal Increased membrane permeability Disruption of H^+^-ATPase Oxidative stress DNA damage	[43]
*Microgramma vacciniifolia* frond lectin (MvFL)	Oligosaccharide chains	Broth microdilution	*C. albicans* URM-5901	MIC: 20 µg/mL (24 h)	For *N. glabratus*: Fungistatic Lysosomal disruption Impairment of mitochondrial membrane potential.	[44]
			*C. krusei* URM-6391	MIC: 1.25 µg/mL (24 h)		
			*C. tropicalis* URM-6551	MIC: 40 µg/mL (24 h)		
			*C. parapsilosis* URM-6951	MIC: 40 µg/mL (24 h)		
			*N. glabratus* URM-4246	MIC: 0.625 µg/mL (24 h)		
*Moringa oleifera* chitin-binding protein 2 (Mo-CBP-2) ^a^	Chitin	Broth microdilution	*C. albicans* ATCC 10231	MIC: 18.9 μM (48 h) MFC: 100 μM (48 h)	Fungistatic Fungicidal Reduced metabolism	[45,46]
			*C. krusei* ATCC 6258	MIC: 20–45 μM (48 h) MFC: 100 μM (48 h)		
			*C. parapsilosis* ATCC 22019			
			*C. tropicalis* (clinical isolate)			
*Mucuna pruriens* seeds	Carrageenan	CLSI M27-A2	*C. parapsilosis* FAMED-FURG	MIC: 1.95 µg/mL (48 h) MFC: 3.9 µg/mL (48 h)	Fungistatic Fungicidal	[31]
*Portulaca elatior* leaf lectin (PeLL)	Chitin, Mannose, Galactose	Broth microdilution	*C. albicans* URM 5901	MIC: 1.48 µg/mL (48 h)	Fungistatic Fungicidal	[47]
			*C. krusei* URM 6351	MIC: 1.48 µg/mL (48 h)		
			*C. tropicalis* URM 6551	MIC: 1.48 µg/mL (48 h)		
			*C. parapsilosis* URM 6345	MIC: 0.74 µg/mL (48 h)		
*Portulaca elatior* root lectin (PeRoL)	Threalose, Glucose, Galactose, Mannose *N*-acetyl-glucosamine Oligosaccharide chains	Broth microdilution	*C. albicans* URM-7098	MIC/MFC: 16 µg/mL (24 h)	Fungistatic and fungicidal effects	[48]
			*C. tropicalis* URM-7092			
			*C. krusei* URM-6391			
			*C. parapsilosis* URM-7087			
*Punica granatum* sarcotesta lectin (PgTeL)	Chitin, Oligosaccharide chains	Broth microdilution	*C. albicans* URM 5901	MIC: 25 µg/mL (24 h) MFC: 50 µg/mL (24 h)	Fungistatic Fungicidal Decrease in ATP levels Lipid peroxidation Cell wall damage	[49]
			*C. krusei* URM 6391	MIC: 12.5 μg/mL (24 h) MFC: 12.5 µg/mL (24 h)		
Q-Griffithsin	Glucose, Mannose, *N*-acetyl-glucosamine	EUCAST EDef 7.1	*Candida albicans* ATCC 32020	MIC: 6 µg/mL (48 h)	Fungistatic Fungicidal Binding to α-mannan Cell wall disruption Oxidative stress	[50]
			*C. glabrata* CDC316	MIC: 95 µg/mL (48 h)		
			*C. parapsilosis* CDC337	MIC: 24 µg/mL (48 h)		
			*C. krusei* CDC397	MIC: 95 µg/mL (48 h)		
			*C. auris* CDC388	MIC: 48 µg/mL (48 h)		
			*C. auris* CDC389	MIC: 95 µg/mL (48 h)		
*Schinus terebinthifolia* leaf lectin (SteLL)	Chitin, Oligosaccharide chains	Broth microdilution	*C. albicans*	MIC: 6.5 µg/mL (24 h) MFC: 26 µg/mL (24 h)	Fungistatic Fungicidal	[51]
Water-soluble *Moringa oleifera* seed lectin (WSMoL)	Chitin, *N*-acetyl-glucosamine, Glucose, Fructose	Broth microdilution	*C. albicans* URM 5901	MIC: 20 µg/mL (48 h) MFC: 80 µg/mL (48 h)	Fungistatic Fungicidal Necrosis Apoptosis Disruption of mitochondrial membrane potential	[52]
			*C. parapsilosis* URM 6345	MIC: 20 µg/mL (48 h) MFC: 80 µg/mL (48 h)		
			*C. krusei* URM 6391	MIC: 20 µg/mL (48 h) MFC: 40 µg/mL (48 h)		
			*N. glabratus* URM 4246	MIC: 20 µg/mL (48 h) MFC: 20 µg/mL (48 h)		

MIC: minimal inhibitory concentration—the breakpoint (the growth inhibition percentage) for determining the MIC is variable and should be consulted in the original articles. MFC: minimal fungicidal concentration. IC_50_: concentration that inhibits growth by 50%. ATCC: American Type Culture Collection. CDC: U.S. Centers for Disease Control and Prevention. CLSI: Clinical and Laboratory Standards Institute. FAMED-FURG: *Faculdade de Medicina da Universidade Federal do Rio Grande*. MTCC: Microbial Type Culture Collection. NGY: Neopeptone Glucose Yeast. NCCPF: National Culture Collection of Pathogenic Fungi. URM: University Recife Mycologia. N.I., not informed in the literature. ^a^ To convert the MIC/MFC values of DvL, MaL, and Mo-CBP_2_ to µg/mL, the native molecular weights of these proteins are 25, 47, and 66 kDa, respectively.

**Table 2:** Representative lectins with activity against *Cryptococcus* spp.: carbohydrate specificity, experimental data, and observed effects.

Lectin	Binding Carbohydrates	Protocol	Target Fungi	Endpoint (Incubation Time)	Effects	Reference
Coagulant *Moringa oleifera* seed lectin (cMoL)	Galactose, Glucose, Raffinose, Lactose, Arabinose, Trehalose, Oligosaccharide chains	Broth microdilution	*C. neoformans* B3501	MIC: 7.5 μg/mL (48 h)	Fungistatic Apoptosis Necrosis	[57]
			*C. neoformans* H99	MIC: 7.5 μg/mL (48 h)		
			*C. gattii* R265	MIC: 7.5 μg/mL (48 h)		
*Myracrodruon urundeuva* heartwood lectin (MuHL)	Chitin, *N*-acetyl-glucosamine, Oligosaccharide chains	Broth microdilution	*C. neoformans* B3501	MIC: 6.25 µg/mL (48 h) MFC: 12.5 µg/mL (48 h)	Fungistatic Fungicidal	[58]
			*C. gattii* R265	MIC: 12.5 µg/mL (48 h) MFC: 25 µg/mL (48 h)		
PgTeL	Chitin, Oligosaccharide chains	Broth microdilution	*C. neoformans* B3501	MIC: 172 µg/mL (24 h)	Fungistatic	[59]
Scytovirin	Tetramannose	Broth microdilution (CLSI M27-A2)	*C. neoformans* serotype A (various strains)	MFC: 0.5–20 µM (48 h)	Fungicidal Decreased capsule size	[60]
			*C. neoformans* serotype D (various strains)	MFC: 20 µM (48 h)		
			*C. gattii* R265	MFC: 10 µM (48 h)		
			*C. gattii* R272	MFC: 0.5 µM (48 h)		
WSMoL	Chitin, *N*-acetyl-glucosamine, Glucose, Fructose	Broth microdilution	*C. neoformans* B3501	MIC: 6.25 μg/mL (48 h)	Fungistatic Lysosomal damage Reduced mitochondrial membrane potential	[61]
			*C. neoformans* H99	MIC: 6.25 μg/mL (48 h)		
			*C. gattii* R265	MIC: 6.25 μg/mL (48 h)		

MIC: minimal inhibitory concentration; the breakpoint (the growth inhibition percentage) for determining the MIC is variable and should be consulted in the original articles. MFC: minimal fungicidal concentration.

**Table 3:** Lectin-antifungal drug interactions against yeast species.

Lectin	Drug	Method	Target Fungi	Synergy Metric	Effect	Reference
ApuL	Fluconazole	Checkerboard	*C. albicans* URM 5901	FICI = 1.0	Additive	[34]
			*C. parapsilosis* URM 6345	FICI = 0.12	Synergism	
cMoL	Fluconazole	Checkerboard	*C. neoformans* B3501	FICI = 2.01	Antagonism	[57]
			*C. neoformans* H99	FICI = 0.26	Synergism	
			*C. gattii* R265	FICI = 108.67	Antagonism	
ConA	Fluconazole	Checkerboard	*C. albicans* ATCC 10231	Reduced antifungal MIC	Additive	[37]
ConM	Fluconazole	Checkerboard	*C. albicans* ATCC 10231	Reduced antifungal MIC	Additive	[37]
Helja	Fluconazole	Checkerboard	*C. albicans* NGY152	FICI = 0.75	Synergism	[41]
MvFL	Fluconazole	Checkerboard	*C. albicans* URM-5901	FICI > 2.0	Antagonism	[44]
			*C. krusei* URM-6391	FICI = 1.0	Additive	
			*C. parapsilosis* URM-6951	FICI = 0.14	Synergism	
			*C. tropicalis* URM-6551	FICI = 1.0	Additive	
			*N. glabratus* URM-4246	FICI > 2.0	Antagonism	
PgTeL	Amphotericin B	Checkerboard	*C. neoformans* B3501	FICI = 2.0	Indifferent	[59]
Scytovirin	Amphotericin B	Checkerboard	*C. neoformans* serotype D 24067	FICI = 0.3	Synergism	[60]
	Fluconazole			FICI = 0.75	Synergism	
	5-flucytosine			FICI = 0.56	Synergism	

FICI: Fractional Inhibitory Concentration Index.

## Data Availability

No new data were created or analyzed in this study. Data sharing is not applicable to this article.
